# Seasonal Heat Stress and the Postpartum Stage Interactively Influence Milk Fatty Acid Composition in Holstein Dairy Cows in Spain

**DOI:** 10.3390/ani15213119

**Published:** 2025-10-27

**Authors:** Elena Niceas Martínez Diez, Rodrigo Muiño Otero, Cristina Castillo Rodríguez, Joaquín Hernández Bermúdez

**Affiliations:** Department of Animal Pathology, Campus Terra-IBADER, University of Santiago de Compostela, 27002 Lugo, Spain; elenaniceas.martinez.diez@usc.es (E.N.M.D.); rodrigo.muino.otero@usc.es (R.M.O.); joaquin.hernandez@usc.es (J.H.B.)

**Keywords:** climate change, heat stress, dairy cows, milk fatty acids, environmental physiology, sustainable livestock systems

## Abstract

**Simple Summary:**

Climate change is causing dairy cows to experience heat stress more often, negatively affecting their health and the quality of the milk they produce. In this study, we evaluated how both heat stress and the weeks after calving influence the types of fat present in cow’s milk. By analyzing milk samples from different seasons and moments after calving, we found that both factors alter the milk’s fatty acid profile in different ways. This information helps farmers and scientists understand how to reduce the effects of climate change on milk quality and maintain productive, healthy herds.

**Abstract:**

Climate change is intensifying heat stress conditions in livestock systems, posing significant challenges to animal welfare, productivity, and food quality. This study aims to investigate the combined effects of seasonal heat stress and postpartum physiology on the milk fatty acid (FA) profile of Holstein dairy cows in Galicia, Spain. Forty milk samples were collected during winter and summer and at 1 week and 1 month postpartum. Fatty acid composition was analyzed via gas chromatography (GC-FID), and heat stress exposure was quantified using the temperature–humidity index (THI). Results revealed that heat stress significantly altered the milk lipid profile, with increased concentrations of short- and medium-chain fatty acids (SMCFA) such as C10:0 and C14:1 (n-5), and conjugated linoleic acids (CLAs), suggesting enhanced de novo lipogenesis and shifts in rumen fermentation. Conversely, unsaturated long-chain fatty acids (LCFAs), including cis-11 C18:1 and cis-13 C18:1, decreased with lactation progression and thermal exposure. Notably, certain LCFAs remained stable under combined seasonal and physiological stress, indicating potential metabolic regulation. These results show how milk composition is sensitive to environmental stress and emphasize the need for climate-resilient management to protect milk quality under global warming.

## 1. Introduction

Milk fat is a highly dynamic and valuable component in ruminants, playing an essential role in biological processes and significantly contributing to the economic value of dairy production. Dairy farming is a key component of Spain’s agri-food sector, producing over 7.5 million tons of cow’s milk annually, mainly concentrated in Galicia, Castilla y León, and Asturias, which together account for more than 60% of national production, according to the Ministerio de Agricultura, Pesca y Alimentación. Galicia alone contributes around 40% of total milk output, with an average herd size of 50–70 cows and a strong prevalence of Holstein breeds under semi-intensive systems. These systems combine housed management with controlled feeding strategies, making them representative of modern dairy production in north-western Spain. In recent years, the increasing demand for milk fat has heightened interest among dairy producers in understanding and optimizing its composition and yield. Recognized as one of the most complex natural fats, milk fat contains over 400 distinct fatty acids (FAs) [1,2].

Milk FA composition results from dietary intake and ruminal microbial activity [2] together with mammary synthesis, with triacylglycerides (TAGs) representing 98% of milk fat [3]. Of these, ~70% are saturated (SFAs), 26% monounsaturated (MUFAs), and 4% polyunsaturated (PUFAs) [1], including approximately 11% short-medium chain FA (SMCFA, <16 carbons) [4]. Around 50% of FA are synthesized de novo in the mammary gland, while ~10% derive from body reserves or dietary lipolysis, rising to 20% under negative energy balance (NEB) [5]. Chain length and origin allow classification: LCFA (>16 carbons) mainly come from diet or mobilization under NEB, whereas SMCFA originate predominantly from mammary de novo synthesis and fat mobilization [6]. Notably, palmitic acid (C16:0) derives approximately half from mammary de novo synthesis and half from preformed long-chain FA uptake (dietary or adipose origin) [5].

Two-key odd-chain FAs, pentadecanoic acid (C15:0) and heptadecanoic acid (C17:0), dominate the milk fat profile and are mainly synthesized by bacterial activity in the rumen [7]. Milk fat composition is influenced by genetic predisposition, lactation stage, mastitis [8], ruminal fermentation, and diet [1,9], with feeding strategies and seasonality being particularly decisive [10,11].

Climate change exerts a direct impact on dairy cows mainly through heat stress, which challenges their ability to maintain thermal homeostasis [12,13]. Elevated ambient temperature and humidity increase core body temperature and respiratory rate, reduce feed intake, and disrupt endocrine and metabolic balance, leading to oxidative stress and immune impairment [14]. These physiological alterations are linked to negative outcomes such as reduced fertility, greater disease susceptibility, and lower welfare. From a productive perspective, heat stress is strongly associated with decreases in dry matter intake, milk yield, and alterations in milk composition [12,13]. It also affects fat production in the mammary gland and alters the fat, protein, and solids-not-fat percentages. In parallel, the metabolism of lipids and insulin regulation is disturbed under heat stress, with increased mobilization of body fat stores and elevated circulating NEFA levels [14].

Seasonal variation in feed availability and quality markedly affects milk fat composition. In summer, grazing on unsaturated fatty acid (UFA)-rich fresh forage reduces SFAs and increases UFAs, whereas winter diets based on conserved forages and concentrates lead to higher SFA levels due to lower availability of unsaturated fats [15]. This impact of season on milk fat composition is less marked in intensive and semi-intensive systems [16], such as those predominant in Spain, where balanced rations are provided year-round. Although diets are not pasture-based and remain relatively consistent, variability still exists between farms due to differences in management, feed availability, and cost.

Regulation of milk fat composition is strongly influenced by the cow’s physiological state. In high-yielding cows under NEB, mobilization of body reserves alters milk fat composition, with de novo FA synthesis in the mammary gland and mobilization of LCFA from adipose tissue playing a key role during peak production [17].

Comparing 1 week and 1 month postpartum is relevant because milk lipid composition changes markedly during early lactation [1,2,16]. At 1 week, milk still retains colostrum and transitional features, with higher levels of bioactive lipids and FA variability [4,7], while by 1 month it has shifted to a stable, mature profile reflecting maternal metabolic adaptations and calf nutritional demands [6,16].

Heat stress, intensified by global warming, challenges dairy cattle by raising body temperature, heart and respiratory rates, and sweating [12]. It reduces dry matter intake (DMI), thereby lowering milk yield and altering fat, protein, lactose, and solids-not-fat percentages [13]. Reduced DMI limits nutrients and precursors (acetate and butyrate) needed for de novo fat synthesis, while physiological changes such as metabolic heat production and nutrient repartitioning further compromise milk production and quality [14,18,19,20].

Although milk lipid composition has been widely studied, most research has examined lactation stage, diet, or season in isolation. Little is known about how these factors interact under real farming conditions, particularly in systems with controlled feeding that may attenuate seasonal effects. This study aims to provide novel insights into the combined influence of physiological and environmental factors on milk fat composition, improving understanding of lipid metabolism in dairy cows under practical conditions.

The objective of this study was to analyze the impact of two key factors on the milk fat composition: (i) postpartum period, by comparing the milk lipid profiles at 1 week and 1 month postpartum and (ii) seasonal variation, by evaluating differences in the milk fat composition between summer and winter, with a focus on potential heat stress effects.

## 2. Materials and Methods

### 2.1. Ethical Approval

All standards for animal handling and care were strictly followed, as indicated in the Spanish Regulations (RD 53/2013), legal provision number 1337, and the European regulation of animals for scientific purposes (Council of Europe, Directive 2010/63/EU). This study is also authorized by the Bioethics Committee of the University of Santiago de Compostela, Spain, according to the relevant Spanish regulations.

### 2.2. Housing System and Ration Composition

The study was conducted in one dairy farm in the Spanish province of Lugo, in Galicia, in 2023. Animals were maintained under intensive rearing conditions, with a total of 227 Holstein lactating cows, and an average daily milk yield of 33.7 kg per cow. Milk fat and protein percentages showed moderate variation, with mean values of 3.80% and 3.36%, respectively. This farm employed natural ventilation systems regulated by adjustable dampers and featured automated milking robots. The barn was oriented East–West, resulting in different shading patterns throughout the day. Concrete flooring was used in all facilities, and sand bedding or similar comfort systems were provided. The most recent quality control data (2023) was used for herd and milk composition characterization for this study.

Cows were housed in a free-stall barn equipped with two automated milking systems (GEA Group, Düsseldorf, Germany) for each lactating group. Consequently, no conventional milking parlor was used, and the environmental conditions during milking were the same as those inside the barn. Average indoor temperatures were 10.3 °C in winter and 20.3 °C in summer, with corresponding mean relative humidity values of 75.8% and 75.7%, respectively. The barn featured natural ventilation through lateral openings, complemented by three axial fans that automatically operated when ambient temperature reached 24 °C and relative humidity exceeded 60%. Although no sprinklers were installed above the animals, a roof-mounted water-spray cooling system was activated under the same temperature and humidity thresholds to limit heat accumulation within the facility.

The facility features two lactation areas, which are equipped with two milking robots each. The nutritional requirements of the animals at lactation are particularly demanding, so a unifeed feed, consisting of maize silage, grass silage, barley straw, and concentrate feed, was provided to the animals to meet these requirements. Additionally, a supplementary isolated concentrate was offered in the milking robot. The unifeed ration was distributed in the barn once a day during early morning hours (8:00–9:00 a.m.) using a mixer wagon. The nutritional data were obtained by Centro Veterinario Meira, Lugo (Meira, Spain; https://centroveterinariomeira.es), using the NDS-Pro software platform (RUM&N Company, Reggio Emilia, Italy; https://www.rumen.it; accessed on 20 January 2025). This platform was developed in collaboration with the Cornell Department of Animal Science and is based on the NRC (2001; Washington, DC, USA) requirements.

The feeding regimen was based on a partial mixed ration (PMR), as part of the concentrate was supplied through the milking robot. The daily ration, calculated per lactating cow on a dry matter basis, consisted of 8.40 kg of corn silage (31.24% of total dry matter), 7.47 kg of grass silage (27.77%), 4.59 kg of robot feed (17.07%), and 6.43 kg of concentrate feed (23.92%). The values in the ‘DM (%)’ column in Table 1 indicate the average daily DM intake per cow for the total ration in the farm where the study was performed.

### 2.3. Technical Design

The study was carried out over two distinct seasonal periods: winter (January to March) and summer (July to September). These periods were selected to represent contrasting climatic conditions.

Multiparous cows (≥2 lactations with 3 or more years) with a body condition score between 2.5 and 3, that were due to calve in January and February or July and August were selected for inclusion using the GEA Group’s DairyPlan version 5.2 breeding program. Selected animals were required to be in good health, showing no signs of disease that could induce metabolic alterations, and an average body weight of approximately 600–650 kg with a body condition score between 2.5 and 3 were included.

A total of 40 milk samples were collected, evenly distributed between the two seasons (*n* = 20 per season). Within each season, *n* = 10 samples corresponded to cows at 1 week postpartum (day 7), and *n* = 10 at 1 month postpartum (day 30). Milk sampling was performed manually on a weekly basis (between 9:00 and 11:00 a.m.) on Mondays. This consistent sampling window was selected based on coordination with farm management practices to minimize disturbance and ensure standardization across all collections. Each sample consisted of 10 mL of milk, collected in sterile tubes. The design with two sampling points was chosen to capture two critical stages of early lactation: the transition period (colostrum/early lactation, day 7) and the establishment of mature milk (day 30). This approach ensured comparability across seasons while minimizing invasive handling of the animals.

### 2.4. Lipid Extraction

Milk samples (10 mL) were collected manually into sterile tubes during the morning sampling session (between 9:00 and 11:00 a.m.), immediately refrigerated (4 °C), and transported to the laboratory within 24 h. Upon arrival, samples were either analyzed directly or stored at −25 °C for no longer than 4 weeks until analysis, as described by Barreiro et al. [21]. Briefly, 10 μL of milk was mixed with 2 mL of H2SO4 (2.5%) in methanol, vortexed for 1 min, and left overnight at 4 °C to ensure efficient lipid extraction and derivatization. This method allowed for simultaneous lipid extraction and methylation in a single step, minimizing sample handling and reducing potential losses. Thereafter, samples were placed in a water bath for 2 h at 60 °C for FA methylation. Methyl nonadecanoate (C19:0) was used as an internal standard to ensure accurate quantification, allowing correction for variations in the extraction efficiency and instrumental response, which is essential for obtaining FA concentrations expressed in milligrams per 100 milliliters. A volume of 1 mL of n-hexane was used to extract fatty acid methyl esters (FAMEs), which were separated via gas chromatography using a 6850 GC system (Agilent Technologies, Palo Alto, CA, USA), equipped with a flame ionization detector (GC–FID) and a DB-Was capillary column (60 m, 0.25 µm id, 0.25 µm film thickness; Agilent Technologies, Inc., Santa Clara, CA, USA). The chromatographic conditions were as follows: The initial oven temperature was set at 35 °C for 2 min and then increased to 100 °C at a rate of 30 °C/min, followed by an increase to 225 °C at 5 °C/min, where it was held for 10 min. The injector and detector temperatures were set at 250 °C and 300 °C, respectively. Helium was utilized as the carrier gas at a flow rate of 1.8 mL/min, with a split ratio of 10:1. Data was collected using integrator software GC ChemStation version B.03.02 (Agilent Technologies). Chromatograms were reviewed to check for proper peak integration and identification, and the percentage of FAs by weight was calculated by dividing the peak area for a particular FA by the total sum of the peak areas for all identified FAs.

A standard mixture of FAMEs (the Supelco 37 Component FAME Mix (Sigma-Aldrich, St. Louis, MO, USA)) was used for calibration to ensure accurate quantification of FAs. The use of this standard mixture allowed proper identification and quantification of individual FAs present in the samples.

All samples were analyzed in duplicate, and mean values were used for the study. A total of 25 FAs were identified. The total SFAs were estimated from the sum of the individual SFAs: C6:0, C8:0, C10:0, C12:0, C14:0, C15:0, C16:0, C17:0, C18:0, and C20:0. For the total MUFAs, the included FAs were C14:1n-5, C16:1n-9, C16:1n-7, C16:1n-5, and C16:1n-13t, cis-9-C18:1, cis-11-C18:1, cis-13-C18:1. Conversely, C18:2 (n-6)9,12t, C18:2 (n-6)9t,12t, C18:2 (n-6)9t,12, C18:3 (n-3) ALA, Ω-3, Ω-6, and CLAs were included for the total PUFAs.

GC-FID is a highly sensitive and accurate technique for the quantification of FAs, allowing detailed identification and quantification of the lipid profile in milk samples. The system has been calibrated using a standard mixture of FAMEs to ensure the accuracy and reproducibility of results.

### 2.5. Temperature and Humidity Measurements

Ambient temperature and relative humidity in the study farms were monitored and recorded using the Multi-Use Compact PDF Temperature and Humidity USB Data Logger (Multicomp Pro; Barcelona, Spain). The device was situated at a height of 2 m on a partition in the lactation area, ensuring it was out of reach of the cows. The logger is capable of measuring temperatures ranging from −30 °C to +70 °C, with an accuracy of ±0.5 °C, and humidity ranging from 0% to 100% RH, with an accuracy of ±3% RH.

THI = 0.81 × Tª + RH/100 × (Tª − 14.4) + 46.4

Being in this formula Tª: Ambient temperature and RH: relative humidity in %.

We classified heat load using the conventional THI categories commonly cited in dairy heat-stress studies to facilitate comparison with previous literature [22]. Nonetheless, recent sources indicate that a THI between 68 and 72 denotes mild heat stress in high-producing cows, whereas values above 79 are associated with severe physiological and performance impacts, depending on management and environmental conditions [23]. We acknowledge this updated context while retaining our original categorization for consistency [24].

### 2.6. Data Analysis

All statistical analyses were performed using R version 4.4.0 in RStudio (R Core Team, 2024; Boston, MA, USA). Prior to analysis, missing values were removed, and categorical variables were properly labeled and converted into factors: postpartum period (1 week postpartum = 1 w, 1 month postpartum = 1 m) and season (winter and summer). Descriptive statistics (mean, standard deviation, minimum, maximum, and sample size) were calculated for each fatty acid across combined groups (1w_Winter, 1w_Summer, 1m_Winter, 1m_Summer), as well as separately for each main factor (period and season). These were computed using functions from the dplyr package and exported to Excel files using openxlsx (version 4.2.8) for integration into the research paper.

A two-way analysis of variance (ANOVA) was performed individually for each fatty acid and lipidic indicator to assess the main effects of lactation stage and season, as well as their interaction. The aov() function was applied iteratively, and *p*-values for main and interaction effects were extracted. Fatty acids showing significant interaction terms (*p* < 0.05) were selected for further biological interpretation. To support interpretation, 95% confidence intervals were calculated for each FA and lipid indicator by group, and interaction plots were generated using ggplot2 (version 3.5.2). These plots represented estimated group means with error bars to visualize how season and postpartum period jointly influenced each variable. All numerical and graphical outputs were systematically exported for reproducibility and documentation.

## 3. Results

### 3.1. Temperature and Humidity Data

**Figure 1:**
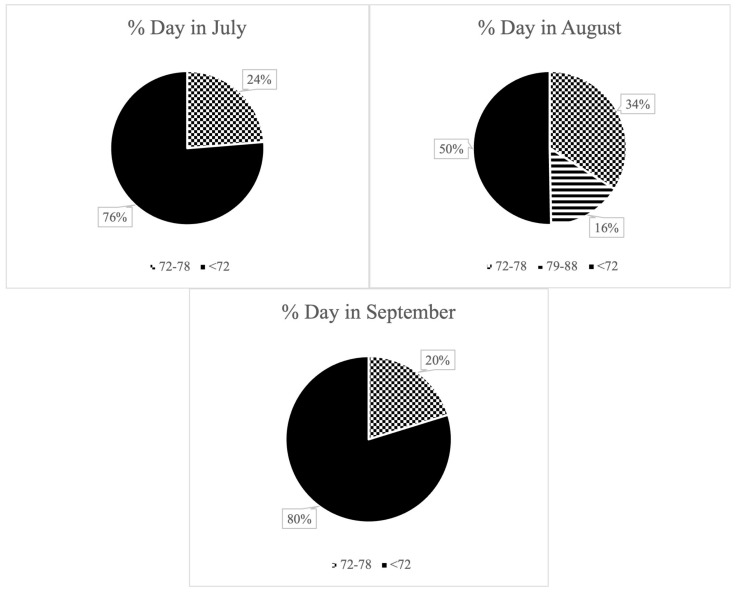
Average daily percentage of time dairy cows spent under varying levels of heat stress during summer months, based on hourly THI records. Segments of the pie chart represent no heat stress (THI ≤ 72, solid black), moderate heat stress (THI “72–78”, dotted pattern), and severe heat stress (THI “79–88”, horizontal stripes, present only in August).

**Figure 2:**
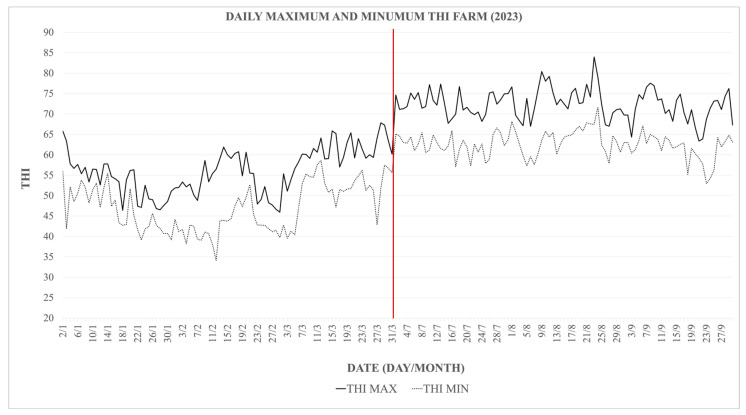
Daily maximum and minimum THI values recorded on the farm throughout 2023. The solid line shows maximum daily THI; the dashed line shows minimum daily THI, illustrating seasonal and daily fluctuations in heat stress exposure.

During the three summer months of the experimental period, the animals were exposed to a THI ranging from 72 to 78 for a total of 49 days and a THI ranging from 79 to 88 for 4 days (Figure 1). However, these values were not sustained throughout the day. In July, days with a THI of 72–78 accounted for an average of 23.7% of the daily time. This proportion significantly increased in August, reaching 33.9%. Additionally, in August, there were days when a THI above 79 was recorded, although this value was maintained for an average of 15.6% of the day. It is important to note that the period during which THI exceeded 79 occurred within the same days in which THI also surpassed 72. Therefore, this moderate heat stress exposure was not isolated, but rather added to the 33.99% of the day already characterized by mild heat stress levels in August. In September, a THI above 79 was not reached; however, THI values ranging from 72 to 78 were documented, representing an average of 20.2% of the daily time on the days when these values were achieved (visually represented in Figure 1). During the winter months, the THI values remained consistently below 68, indicating the absence of heat stress conditions during this period (Figure 2).

Each pie chart represents the average daily percentage of time that cows were exposed to the following THI ranges: <72: no heat stress; solid black; “72–78”: moderate heat stress; dotted pattern; “79–88”: severe heat stress; horizontal stripes; only present in August. Values were calculated from hourly THI records.

### 3.2. Effects of Season on Milk Fatty Acid Profile

Table 2 summarizes the seasonal comparison between winter and summer, and reveals significant variations in several individual fatty acids, while total lipid classes remained statistically unaffected.

Among the SMCFA, which are primarily synthesized de novo, a notable increase was observed in C10:0 (from 102.26 ± 17.62 to 128.25 ± 22.10 mg/100 mL milk; *p* = 0.0286) and C14:1(n-5) (from 28.05 ± 4.51 to 38.86 ± 7.89 mg/100 mL milk; *p* = 0.0034) during summer. About LCFA, several components linked to dietary intake or microbial hydrogenation exhibited significant differences. Specifically, C15:0 increased markedly in summer (25.75 ± 4.22 vs. 42.06 ± 7.21 mg/100 mL milk; *p* < 0.0001). In contrast, cis-11 C18:1 decreased significantly in summer (7.40 ± 1.17 vs. 4.08 ± 1.40 mg/100 mL milk; *p* = 0.0002).

Other major fatty acid groups, such as SFA, MUFA, PUFA, omega-3, omega-6, and total fatty acid content, did not show statistically significant differences between seasons (*p* > 0.05). However, CLAs were significantly higher in summer (32.16 ± 5.93 vs. 24.02 ± 3.85 mg/100 mL milk; *p* = 0.0139). Within the omega-6 fraction, C18:2 (n-6) 9t,12t showed a significant decrease in summer compared to winter (1.69 ± 0.16 vs. 2.36 ± 0.20 mg/100 mL milk; *p* = 0.012).

### 3.3. Effects of Postpartum Period on Milk Fatty Acid Profiles

Table 3 presents the results of the two-way ANOVA examining the effects of postpartum period (1 week vs. 1 month) and table between seasons (winter vs. summer) on total FA content.

The two-way ANOVA revealed significant effects of lactation stage on several SMCFAs, primarily associated with de novo synthesis and fat mobilization. Concentrations of C6:0, C8:0, C10:0, C12:0, and C14:1(n-5) were significantly higher at 1 month postpartum compared to 1 week (*p* < 0.05). Among the LCFAs, derived from dietary origin, ruminal biohydrogenation, or body fat mobilization, significant increases were observed for C15:0 at 1 month postpartum (*p* = 0.0177), whereas C16:1(n-5), cis-11 C18:1, and cis-13 C18:1 showed significant decreases (*p* = 0.0293, *p* = 0.0073, and *p* = 0.0259, respectively). Notably, the concentration of cis-11 C18:1 dropped from 6.88 ± 1.47 mg/100 mL milk to 4.60 ± 1.36 mg/100 mL milk.

In contrast, no significant differences were found between time points for SFA, MUFA, PUFA, or lipid classes such as omega-3, omega-6, and CLA (*p* > 0.05). Likewise, total fatty acid content remained statistically unchanged between 1 week and 1 month postpartum (2753.93 ± 442.18 vs. 2919.80 ± 471.65 mg/100 mL milk, *p* = 0.5337). However, within the omega-3 fraction, α-linolenic acid (C18:3 n-3, ALA) showed a significant increase from 1 week to 1 month postpartum (10.10 ± 0.97 vs. 15.57 ± 1.05 mg/100 mL milk, *p* = 0.0007).

### 3.4. Effects of Postpartum Period and Season on Milk Fatty Acid Profiles (Interaction)

The two-way ANOVA revealed several significant interactions between lactation stage and season affecting individual fatty acid concentrations in milk (Table 4).

**Figure 3:**
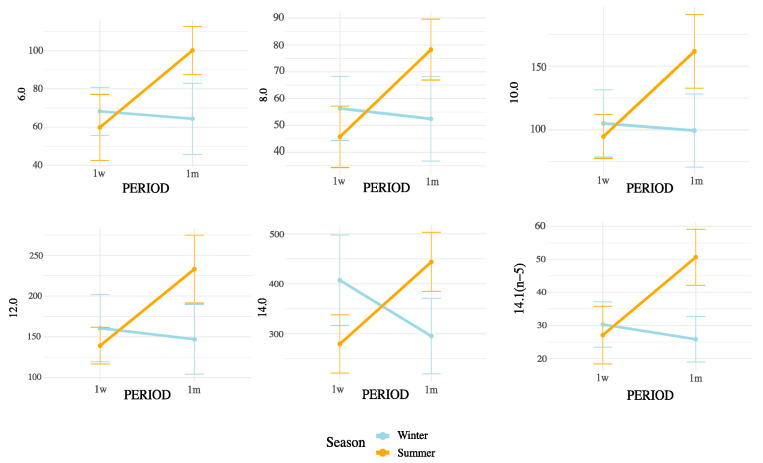
Interaction effects of postpartum period and season on SMCFA concentrations in milk. Values are means ± 95% confidence intervals at 1 week (1w) and 1 month postpartum (1m), with winter (blue line) and summer (orange line) seasons plotted. Results indicate differences in metabolic responses influencing milk fatty acid synthesis across seasons and lactation stages.

**Figure 4:**
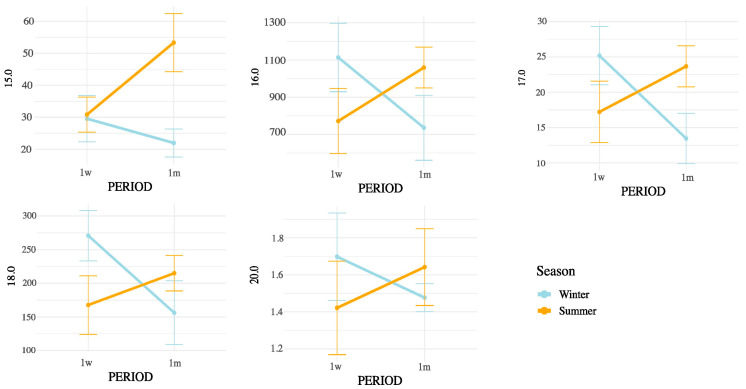
Interaction between postpartum period and season on saturated LCFA concentrations in milk. Values are means ± 95% confidence intervals at 1 week (1w) and 1 month postpartum (1m), with winter (blue line) and summer (orange line) seasons plotted. Results indicate differences in metabolic responses influencing milk fatty acid synthesis across seasons and lactation stages.

Notably, all SMCFA (C6:0, C8:0, C10:0, C12:0, C14:0, and C14:1(n-5)) showed highly significant interaction effects (*p* < 0.01), suggesting that their concentrations were influenced not only by postpartum stage or season independently, but by their combination. Among LCFAs, strong interaction effects were observed for C15:0, C16:0, C16:1(n-9), C16:1(n-7), C16:1(n-13)t, C17:0, C18:0, and C20:0 (all *p* < 0.05). In contrast, some unsaturated LCFAs such as cis-9 C18:1, cis-11 C18:1, and cis-13 C18:1 showed no significant interaction. Importantly, the interaction term was also significant for broader lipidic indicators, including total fatty acids (*p* = 0.0004), SFA (*p* = 0.0001), MUFA (*p* = 0.0147), PUFA (*p* = 0.0021), omega-3 (*p* = 0.0005), omega-6 (*p* = 0.0024), and CLAs (*p* = 0.0104). These results underscore that both the metabolic adaptations during early lactation and the exposure to summer heat stress may synergistically affect the lipid composition of milk. These interactions are represented in Figure 3 (SMCFA) and Figure 4 (LCFA).

**Figure 5:**
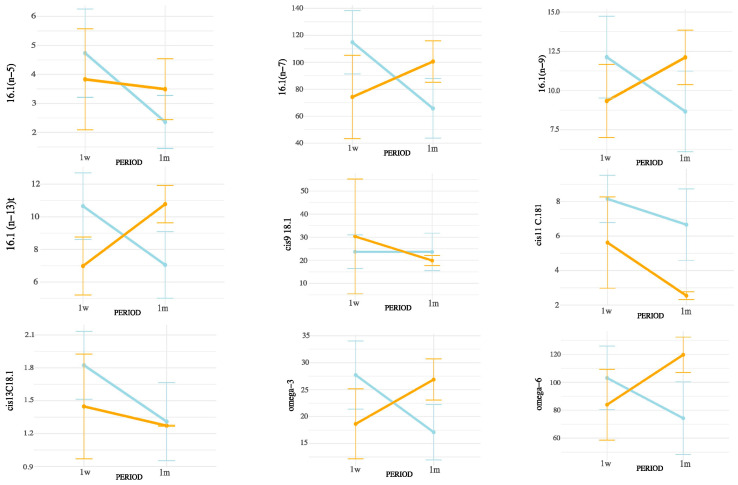
Interaction between postpartum period and season on unsaturated LCFA concentrations in milk. Values are mean ± 95% confidence intervals at 1 week (1w) and 1 month postpartum (1m), with winter (blue line) and summer (orange line) seasons plotted. Results indicate differences in metabolic responses influencing milk fatty acid synthesis across seasons and lactation stages.

**Figure 6:**
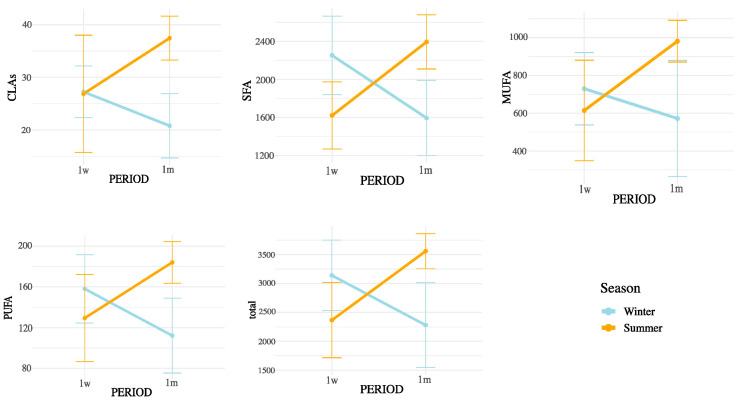
Interaction between postpartum period and season on CLAs, SFA, MUFA, PUFA, and total FA concentrations in milk. Values are mean ± 95% confidence intervals at 1 week (1w) and 1 month postpartum (1m), with winter (blue line) and summer (orange line) seasons plotted. Results indicate differences in metabolic responses influencing milk fatty acid synthesis across seasons and lactation stages.

Figure 4, Figure 5 and Figure 6 display the mean concentrations (±95% confidence interval) of individual LCFAs (C15:0, C16:0, C18:0, cis-9-C18:1, cis-11-C18:1, cis-13-C18:1, C20:0, omega-3, and omega-6) and total lipid fractions (SFA, MUFA, PUFA, and total fat) in bovine milk, stratified by postpartum stage on the x axis (1 week vs. 1 month) and season (winter vs. summer). Notable interaction effects highlight how dietary intake, ruminal biohydrogenation, and adipose mobilization may vary in response to environmental and physiological factors.

## 4. Discussion

It is well known that heat stress can alter milk fat composition, compromising both the nutritional quality and technological properties of milk, ultimately affecting dairy farm profitability [25,26]. In the present study, the interaction between season and postpartum stage was the main driver of variation in milk fatty acid composition. Our results confirmed that the interaction between season and postpartum stage exerted a marked influence on the milk fatty acid profile, with distinct responses across combinations (W1w, W1m, S1w, S1m). Summer and early-lactation conditions jointly modified de novo synthesized SMCFA and CLA concentrations, while certain unsaturated LCFAs decreased, indicating non-additive and stage-dependent thermal effects [26,27,28].

These findings are particularly relevant in the context of global climate change, which is expected to increase the frequency and intensity of heat stress events in livestock systems. Understanding how thermal stress alters milk composition is essential for developing climate-resilient strategies in dairy production. Importantly, the interactive effect observed here shows that environmental and physiological factors do not act independently but mutually modulate the mammary lipid response throughout early lactation [27,28].

Figure 1 and Figure 2 illustrate the seasonal variation in heat stress exposure, with August showing the highest proportion of the day under elevated THI levels (33.9–49.5%), compared to July (23.7%) and September (20.2%). However, this exposure was not continuous throughout the day. Cows experienced intermittent periods of thermal relief (76.3%, 50.5–66.1%, and 79.8% of the day in July, August, and September, respectively), which may have partially mitigated the adverse effects of heat stress on milk production and composition.

According to the interaction patterns (Table 4), the combined effect of season and postpartum revealed that SMCFA, such as C10:0 and C14:1(n-5), were highest in the summer–one-month group (S1m), suggesting that thermal stress amplified the activation of mammary de novo lipogenesis later in early lactation [26]. LCFAs, such as C15:0 and CLAs, also peaked in S1m, possibly reflecting altered rumen microbial activity and biohydrogenation under heat stress [27,28]. This seasonal rise in CLA likely results from altered ruminal biohydrogenation dynamics and sustained Δ^9^-desaturase activity under thermal load. Heat stress may favor Butyrivibrio spp. capable of producing vaccenic acid (C18:1 trans-11), the main precursor of cis-9, trans-11 CLA, while the mammary Δ^9^-desaturase remains active or upregulated to convert this substrate into CLA, helping maintain cellular membrane fluidity and antioxidant balance. Similar patterns have been reported in heat-stressed dairy cows [18]. In contrast, minor unsaturated fatty acids such as cis-11 C18:1, cis-13 C18:1, and C16:1 (n-5) showed lower values in summer and early postpartum but no significant season × postpartum interaction, indicating that their variation was mainly driven by individual factors rather than combined effects [25,28]. Nevertheless, the overall interactive pattern highlights that heat load and physiological recovery jointly modulate desaturase activity and microbial lipid transformation [26,29]. These results indicate that seasonal heat stress does not uniformly affect all FAs but selectively alters metabolic and microbial pathways [29].

Although the postpartum stage alone showed progressive recovery of de novo synthesis, its interaction with season revealed that this metabolic adjustment was strongly modulated by the thermal environment. For instance, the relative increase in SMCFA from 1 week to 1 month postpartum was much more pronounced under summer than winter conditions, suggesting that heat exposure accelerates metabolic adaptation in the mammary gland [30,31,32,33,34]. Alterations in milk lipid profiles under heat stress may not only affect technological properties of dairy products but also their nutritional value, with potential implications for food quality and consumer health. This highlights the need for adaptive feeding and housing strategies to maintain milk quality under increasingly variable climatic conditions [18,26,28].

The interaction analysis (Table 4) confirmed that not all effects were additive. While SMCFA and CLA increased sharply in S1m, several unsaturated LCFAs (cis-9 C18:1, cis-11 C18:1, cis-13 C18:1, C18:2 (n-6) 9,12t, C18:2 (n-6) 9t,12t, C18:2 (n-6) 9t,12, and C18:3 (n-3) ALA) remained stable across combinations, revealing compensatory or threshold-type regulatory responses [6,13,25,26,27,28,29]. This finding suggests that the mammary gland dynamically prioritizes lipid homeostasis when facing simultaneous environmental and physiological stress. Taken together, these results demonstrate that interpreting either seasonal or postpartum effects in isolation would underestimate the true complexity of milk FA regulation under field conditions.

Notably, among the individual fatty acids analyzed, α-linolenic acid (C18:3 n-3, ALA) showed a significant increase from 1 week to 1 month postpartum, whereas C18:2 (n-6) 9t,12t decreased during summer compared to winter. These opposite trends likely reflect the differential sensitivity of ruminal biohydrogenation and mammary desaturation pathways to energy balance and heat load. The postpartum rise in ALA suggests enhanced dietary uptake and transfer efficiency as metabolic stability is restored [27,28,31], while the summer decline in trans-linoleic acid is consistent with altered rumen fermentation and reduced production of trans intermediates under heat stress [10,25,30].

From an environmental perspective, the study underscores the vulnerability of intensive dairy systems to climate variability. Incorporating heat mitigation technologies, optimizing feeding strategies, and selecting thermotolerant breeds are key components of sustainable livestock management. Future research should explore the integration of environmental indicators, such as carbon footprint or water use, to assess the broader impact of climate stress on dairy production systems. Overall, our findings provide novel evidence that the interaction of season and postpartum stage must be considered when designing feeding and management strategies to maintain milk quality under climate change scenarios.

Although THI was used to characterize the environmental conditions during the summer period, it was not treated as a continuous independent variable in our statistical models. This decision was based on the nature of the data, which reflected group-level exposure rather than individual cow-level THI records. Moreover, THI values exhibited substantial intra-day variability, and their aggregation into daily averages could introduce confounding effects when correlated with milk fatty acid concentrations. Instead, our factorial design (season × postpartum period) allowed for a more biologically meaningful interpretation of the combined effects of environmental and physiological stressors. The significant interaction terms observed in the ANOVA (Table 4 and Table S1; Figure 3, Figure 4, Figure 5 and Figure 6) already capture the influence of thermal stress as embedded within seasonal variation. Future studies with individual-level THI monitoring may be better suited to explore direct correlations or regression models linking THI and milk lipid profiles.

## 5. Conclusions

Under field conditions in northwestern Spain, milk fatty acid composition was mainly influenced by the interaction between season and postpartum stage, evidencing non-additive responses of mammary lipid metabolism. Summer and late-early lactation (S1m) were associated with higher C10:0, C14:1(n-5), C15:0, and CLA, together with a reduction in cis-11 C18:1, indicating that heat stress promotes synthesis but modifies ruminal biohydrogenation dynamics.

Across the postpartum period, several short- and medium-chain fatty acids increased from 1 week to 1 month, while minor unsaturated FAs such as cis-11 and cis-13 C18:1 decreased. Although global lipid classes (SFA, MUFA, PUFA, ω-3, and ω-6) remained stable, α-linolenic acid (C18:3 n-3 and ALA) significantly rose with advancing postpartum stage, and C18:2 (n-6) 9t,12t declined in summer, reflecting differential sensitivity of ruminal and mammary lipid pathways to energy balance and heat load. These results highlight that thermal and physiological factors act synergistically, not independently, in shaping milk fat composition.

## Figures and Tables

**Table 1:** Physicochemical composition of the ration.

Components		
Nutrients	Unity	DM
DM	%	44.486
CP	%	16.520
CF	%	-
aNDFom	%	29.123
peNDF	%	18.688
NFC	%	42.76
EE	%	4.054
*C12:0*	g/day	0.905
*C14:0*	g/day	9.155
*C16:0*	g/day	258.190
*C16:1*	g/day	1.165
*C18:0*	g/day	30.255
*C18:1t*	g/day	0.505
*C18:1c*	g/day	185.565
*C18:2*	g/day	254.68
*18:3-ALA*	g/day	44.110
*20:5-EPA*	g/day	-
*22:5-DPA*	g/day	-
*22:6-DHA*	g/day	-
*Ration*	g/day	802.65
*Total RUFAL*	g/day	479.3
*Total UFA*	g/day	481.5
*Total PUFA*	g/day	293.9
Ω*-6/* Ω*-3*	g/day	25.01
Starch	%	26.079
Sugars	%	6.528
Ashes	%	7.127

DM: Dry matter; CP: Crude protein; CF: Crude fiber; aNFDom: Ash-free neutral detergent fibre with thermostable amylase; peNDF: Physically effective neutral detergent fibre; NFC: Non-fibrous carbohydrate; EE: Ether extract; C12:0: Lauric acid; C14:0: Myristic acid; C16:0: Palmitic acid; C16:1: Palmitoleic acid; C18:0: Stearic acid; C18:1: Oleic acid; C18:2: Linoleic acid; 18:3-ALA: Alpha-linolenic acid; Total RUFAL: Total unsaturated fatty acid in the rumen; Total UFA: Total unsaturated fatty acid; Total PUFA: Total polyunsaturated fatty acid; Ω-6/Ω-3: Omega-6 to omega-3 ratio.

**Table 2:** Two-way ANOVA results for the effect of season on milk fatty acid composition (mg/100 mL milk): winter and summer means (±95% confidence interval).

FA Origin	FA	Mean-Winter	SEM	Mean-Summer	SEM	*p*-Value
Short and medium chain fatty acid (‘de novo’ and fat mobilization)	6:0	66.348	4.854	80.027	6.525	0.054
	8:0	54.401	4.272	62.004	5.087	0.185
	10:0	102.264	8.419	128.254	10.558	0.029 *
	12:0	153.841	12.846	186.126	14.831	0.062
	14:0	351.151	28.41	361.566	25.961	0.746
	14:1(n-5)	28.052	2.153	38.856	3.77	0.003 *
Long chain fatty acid (Diet, ruminal hydrogenation and fat mobilization)	15:0	25.752	2.017	42.062	3.444	0.000 *
	16:0	924.559	69.735	915.374	55.356	0.9
	16:1(n-9)	10.398	0.882	10.723	0.701	0.754
	16:1(n-7)	90.308	8.925	87.421	8.015	0.784
	16:1(n-5)	3.547	0.469	3.66	0.44	0.851
	16:1(n-13)t	8.389	0.768	8.417	0.648	0.972
	17:0	19.316	1.782	20.437	1.347	0.506
	18:0	213.583	18.502	191.442	12.22	0.213
	cis-9 18:1	23.657	2.353	25.111	5.505	0.811
	cis-11 C18:1	7.402	0.561	4.083	0.67	0.000 *
	cis-13C18:1	1.566	0.117	1.36	0.105	0.172
	C18:2(n-6)9,12t	2.586	0.23	2.569	0.17	0.952
	C18:2(n-6)9t,12t	2.363	0.201	1.691	0.164	0.012 *
	C18:2(n-6)9t,12	2.161	0.163	2.133	0.089	0.882
	C18:3(n-3)ALA	12.676	1.312	12.997	1.053	0.828
	20:0	1.587	0.059	1.532	0.075	0.543
	total	2710.611	227.686	2963.113	206.438	0.345
	SFA	1923.676	143.981	2007.637	131.639	0.605
	MUFA	651.049	79.925	797.985	74.942	0.159
	PUFA	135.11	11.92	156.731	11.953	0.163
	omega-3	22.402	2.136	22.756	1.878	0.886
	omega-6	88.689	8.128	101.817	7.339	0.192
	CLAs	24.018	1.839	32.158	2.831	0.014 *

* *p* < 0.05; Values in bold superscript denote significant differences.

**Table 3:** Two-way ANOVA results for the effect of period on milk fatty acid composition (mg/100 mL milk): 1 week pp and 1 month pp means (±95% confidence interval).

FA Origin	FA	Mean-1 Week	SEM	Mean-1 Month	SEM	*p*-Value
Short and medium chain fatty acid (‘de novo’ and fat mobilization)	6:0	64.09	4.696	82.285	6.348	0.012 *
	8:0	51.054	3.761	65.351	5.112	0.016 *
	10:0	99.947	6.898	130.572	11.307	0.011 *
	12:0	149.927	10.383	190.04	16.195	0.022 *
	14:0	343.237	27.379	369.479	26.763	0.416
	14:1(n-5)	28.677	2.42	38.232	3.696	0.009 *
Long chain fatty acid (Diet, ruminal hydrogenation and fat mobilization)	15:0	30.182	1.957	37.632	4.2	0.018 *
	16:0	942.527	67.31	897.406	57.838	0.537
	16:1(n-9)	10.733	0.817	10.388	0.775	0.74
	16:1(n-7)	94.551	9.553	83.178	7.032	0.283
	16:1(n-5)	4.282	0.509	2.926	0.326	0.029 *
	16:1(n-13)t	8.354	0.739	8.452	0.681	0.905
	17:0	21.192	1.579	18.561	1.533	0.124
	18:0	219.369	17.105	185.656	13.493	0.061
	cis-9 18:1	27.022	5.629	21.746	1.865	0.387
	cis-11 C18:1	6.884	0.703	4.601	0.65	0.007 *
	cis-13C18:1	1.635	0.13	1.291	0.077	0.026 *
	C18:2(n-6)9,12t	2.451	0.247	2.705	0.138	0.388
	C18:2(n-6)9t,12t	1.799	0.172	2.255	0.21	0.083
	C18:2(n-6)9t,12	2.126	0.154	2.167	0.104	0 83
	C18:3(n-3) ALA	10.102	0.968	15.571	1.053	0.0007 *
	20:0	1.56	0.081	1.559	0.051	0.995
	total	2753.929	211.263	2919.795	225.343	0.534
	SFA	1936.97	137.19	1994.344	139.059	0.724
	MUFA	672.234	71.796	776.8	84.44	0.313
	PUFA	143.757	12.132	148.084	12.231	0.777
	omega-3	23.174	2.214	21.985	1.775	0.631
	omega-6	93.527	7.644	96.979	8.107	0.728
	CLAs	27.056	2.618	29.12	2.485	0.516

* *p* < 0.05; Values in bold superscript denote significant differences.

**Table 4:** Two-way ANOVA results for the interaction between lactation stage and season on milk fatty acid profile.

FA Origin	Variable	W/1w	SEM	W/1m	SEM	S/1w	SEM	S/1m	SEM	*p*-Value
Short and medium chain fatty acid (‘de novo’ and fat mobilization)	6:0	68.306	5.552	64.39	8.235	59.875	7.637	100.179	5.585	0.003 *
	8:0	56.348	5.286	52.453	6.948	45.76	5.054	78.248	5.009	0.003 *
	10:0	105.069	11.691	99.46	12.683	94.825	7.643	161.683	12.802	0.003 *
	12:0	160.679	18.182	147.003	18.863	139.175	9.947	233.077	18.438	0.003 *
	14:0	407.04	39.959	295.261	33.421	279.434	25.76	443.698	26.135	0.000 *
	14:1(n-5)	30.291	3.023	25.813	3.052	27.062	3.874	50.651	3.756	0.000 *
Long chain fatty acid (Diet, ruminal hydrogenation and fat mobilization)	15:0	29.55	3.196	21.954	1.938	30.815	2.423	53.31	4.012	0.000 *
	16:0	1113.366	81.433	735.752	77.325	771.688	77.539	1059.061	48.352	0.000 *
	16:1(n-9)	12.13	1.154	8.666	1.136	9.337	1.028	12.11	0.767	0.005 *
	16:1(n-7)	114.841	10.349	65.775	9.773	74.26	13.667	100.581	6.782	0.001 *
	16:1(n-5)	4.734	0.674	2.361	0.403	3.83	0.771	3.491	0.465	0.097
	16:1(n-13)t	10.243	0.928	6.535	0.93	6.465	0.808	10.369	0.524	0.000 *
	17:0	25.17	1.819	13.462	1.575	17.213	1.923	23.66	1.288	0.000 *
	18:0	270.954	16.553	156.213	20.975	167.785	19.237	215.099	11.665	0.000 *
	cis-9 18:1	23.701	3.224	23.613	3.603	30.343	10.997	19.878	0.964	0.395
	cis-11 C18:1	8.148	0.608	6.657	0.914	5.619	1.167	2.546	0.1	0.331
	cis-13C18:1	1.823	0.137	1.31	0.157	1.447	0.211	1.272	0.002	0.263
	C18:2(n-6)9,12t	2.5	0.396	2.672	0.255	2.401	0.317	2.737	0.124	0.78
	C18:2(n-6)9t,12t	2.093	0.084	2.634	0.307	1.505	0.214	1.876	0.246	0.742
	C18:2(n-6)9t,12	2.061	0.27	2.26	0.192	2.191	0.161	2.074	0.084	0.409
	C18:3(n-3)ALA	10.028	1.437	15.323	1.909	10.175	1.375	15.818	1.011	0.9064
	20:0	1.698	0.104	1.477	0.033	1.422	0.112	1.642	0.092	0.020 *
	total	3141.091	268.756	2280.132	324.766	2366.768	287.936	3559.459	134.173	0.000 *
	SFA	2252.177	182	1595.176	174.426	1621.763	155.767	2393.511	125.755	0.000 *
	MUFA	729.799	84.703	572.298	135.715	614.668	117.699	981.301	48.913	0.015 *
	PUFA	158.051	14.785	112.169	16.261	129.462	18.905	183.999	8.978	0.002 *
	omega-3	27.709	2.803	17.096	2.267	18.639	2.876	26.873	1.689	0.001 *
	omega-6	103.1	10.058	74.278	11.472	83.953	11.187	119.68	5.6	0.002 *
	CLAs	27.242	2.169	20.795	2.696	26.87	4.922	37.445	1.844	0.010 *

W1w: Winter—1 week postpartum; W1m: Winter—1 month postpartum; S1w: Summer—1 week postpartum; S1m: Summer—1 month postpartum; * *p* < 0.05; values in bold superscript denote significant differences.

## Data Availability

The data supporting the findings of this study are available from the corresponding author upon reasonable request.

## Supplementary Materials

The following supporting information can be downloaded at: https://www.mdpi.com/article/10.3390/ani15213119/s1, Table S1: Mean (± SEM) concentrations of milk fatty acids (mg/100 mL milk) by postpartum period (1 w, 1 m), season (Winter, Summer), and their interaction in Holstein cows under oceanic climate conditions.
