# Supplementary material for: Seasonal Heat Stress and the Postpartum Stage Interactively Influence Milk Fatty Acid Composition in Holstein Dairy Cows in Spain
Source: Animals (Basel). 2025 Oct 27;15(21):3119. doi: 10.3390/ani15213119 (PMC12609924; doi:10.3390/ani15213119)
Supplement: Supplementary file 1 [file animals-15-03119-s001.zip › animals-3906557-supplementary.pdf]

Table S1: Mean (± SEM) concentrations of milk fatty acids (mg/100 mL milk) by postpartum period (1w, 1m), season (Winter, Summer), and their interaction in Holstein cows under oceanic climate conditions.

FA	Post-partum		Season		Interaction				SEM	p-value Pp	p-value Season	p-value interaction
	1w	1m	W	S	W1w	W1m	S1w	S1m				
6:00	64.090	82.285	66.348	80.027	68.306	64.390	59.875	100.179	4.161	0.012*	0.054	0.003*
8:00	51.054	65.351	54.401	62.004	56.348	52.453	45.760	78.248	3.335	0.016*	0.185	0.003*
10:00	99.947	130.572	102.264	128.254	105.069	99.460	94.825	161.683	6.982	0.011*	0.029*	0.003*
12:00	149.927	190.040	153.841	186.126	160.679	147.003	139.175	233.077	10.023	0.022*	0.062	0.003*
14:00	343.237	369.479	351.151	361.566	407.040	295.261	279.434	443.698	19.013	0.416	0.746	0.000*
14:1(n-5)	28.677	38.232	28.052	38.856	30.291	25.813	27.062	50.651	2.311	0.009*	0.003*	0.000*
15:00	30.182	37.632	25.752	42.062	29.550	21.954	30.815	53.310	2.363	0.018*	0.000*	0.000*
16:00	942.527	897.406	924.559	915.374	1113.366	735.752	771.688	1059.061	43.949	0.537	0.9	0.000*
16:1(n-9)	10.733	10.388	10.398	10.723	12.130	8.666	9.337	12.110	0.557	0.740	0.754	0.005*
16:1(n-7)	94.551	83.178	90.308	87.421	114.841	65.775	74.260	100.581	5.925	0.283	0.784	0.001*
16:1(n-5)	4.282	2.926	3.547	3.660	4.734	2.361	3.830	3.491	0.318	0.029*	0.851	0.097
16:1(n-13)t	8.354	8.452	8.389	8.417	10.243	6.535	6.465	10.369	0.496	0.905	0.972	0.000*
17:00	21.192	18.561	19.316	20.437	25.170	13.462	17.213	23.660	1.106	0.124	0.506	0.000*
18:00	219.369	185.656	213.583	191.442	270.954	156.213	167.785	215.099	11.086	0.061	0.213	0.000*
cis-9 18:1	27.022	21.746	23.657	25.111	23.701	23.613	30.343	19.878	2.957	0.387	0.811	0.395
cis-11 C18:1	6.884	4.601	7.402	4.083	8.148	6.657	5.619	2.546	0.507	0.007*	0.000*	0.331
cis-13C18:1	1.635	1.291	1.566	1.360	1.823	1.310	1.447	1.272	0.079	0.026*	0.172	0.263
C18:2(n-6)9,12t	2.451	2.705	2.586	2.569	2.5	2.672	2.401	2.737	0.092	0.388	0.952	0.78
C18:2(n-6)9t,12t	1.799	2.255	2.363	1.691	2.093	2.634	1.505	1.876	0.139	0.083	0.012*	0.742
C18:2(n-6)9t,12	2.126	2.167	2.161	2.133	2.061	2.26	2.191	2.074	0.141	0 83	0.882	0.409
C18:3(n-3)ALA	10.102	15.571	12.676	12.997	10.028	15.323	10.175	15.818	0.831	0.0007*	0.828	0.9064
20:00	1.560	1.559	1.587	1.532	1.698	1.477	1.422	1.642	0.047	0.995	0.543	0.020*
total	2753.929	2919.795	2710.611	2963.113	3141.091	2280.132	2366.768	3559.459	153.028	0.534	0.345	0.000*
SFA	1936.970	1994.344	1923.676	2007.637	2252.177	1595.176	1621.763	2393.511	96.520	0.724	0.605	0.000*
MUFA	672.234	776.800	651.049	797.985	729.799	572.298	614.668	981.301	55.340	0.313	0.159	0.015*
PUFA	143.757	148.084	135.110	156.731	158.051	112.169	129.462	183.999	8.510	0.777	0.163	0.002*
omega-3	23.174	21.985	22.402	22.756	27.709	17.096	18.639	26.873	1.404	0.631	0.886	0.001*
omega-6	93.527	96.979	88.689	101.817	103.100	74.278	83.953	119.680	5.506	0.728	0.192	0.002*
CLAs	27.056	29.120	24.018	32.158	27.242	20.795	26.870	37.445	1.789	0.516	0.014*	0.010*

Values represent means and their standard error of the mean (SEM); Postpartum period (Pp): 1w = 1 week postpartum, 1m = 1 month postpartum; Season: W = Winter, S = Summer; Interactions combine both effects (W1w, W1m, S1w, S1m); p-values correspond to the main effects of postpartum period, season, and their interaction from the two-way model ANOVA; * : $p < 0.05$; FA abbreviations: SFA (saturated fatty acids), MUFA (monounsaturated fatty acids), PUFA

(polyunsaturated fatty acids), CLA (conjugated linoleic acids); Short- and medium-chain FA (C6:0–C14:0) are mainly of de novo and adipose origin, while long-chain FA (≥ C16:0) derive from diet, ruminal biohydrogenation, and lipid mobilization.
